# A Sensitive SERS Method for Determination of Pymetrozine in Apple and Cabbage Based on an Easily Prepared Substrate

**DOI:** 10.3390/foods10081874

**Published:** 2021-08-13

**Authors:** Ting-Tiao Pan, Mei-Ting Guo, Wang Guo, Ping Lu, De-Yu Hu

**Affiliations:** 1 State Key Laboratory Breeding Base of Green Pesticide and Agricultural Bioengineering, Key Laboratory of Green Pesticide and Agricultural Bioengineering, Ministry of Education, Center for R&D of Fine Chemicals of Guizhou University, Guiyang 550025, China; pantingtiaos@163.com (T.-T.P.); gmt1725@163.com (M.-T.G.); herrguowang@163.com (W.G.); plu@gzu.edu.cn (P.L.); 2 College of Biological Sciences and Agriculture, Qiannan Normal University for Nationalities, Duyun 558000, China

**Keywords:** insecticide, pymetrozine, residual detection, food, surface-enhanced Raman spectroscopy

## Abstract

Residual pesticides are one of the major food safety concerns around the world. There is a demand for simple and reliable methods to monitor pesticide residues in foods. In this study, a sensitive method for determination of pymetrozine in apple and cabbage samples using surface-enhanced Raman spectroscopy (SERS) based on decanethiol functionalized silver nanoparticles was established. The proposed method performed satisfactorily with the linear detection range of 0.01–1.00 mg/L and limit of detection (LOD) of 0.01 mg/L in methanol. In addition, it was successfully used to detect pymetrozine in apple and cabbage samples, the LOD was 0.02 and 0.03 mg/L, respectively, and the recoveries of spiked cabbage and apple ranged 70.40–104.00%, with relative standard deviations below 12.18% and 10.33% for intra-day and inter-day tests. Moreover, the results of the correlation test with real cabbage samples of liquid chromatography-tandem mass spectrometry showed that they were highly correlated (slope = 0.9895, *R*^2^ = 0.9953). This study provides a sensitive approach for detection of pymetrozine in apple and cabbage, which has great potential for determination of pymetrozine residues in food products.

## 1. Introduction

In modern agriculture, pesticides are widely used to improve the yield of crops and quality of farm products [1]. However, the overuse of pesticides will lead to high pesticide residues, which are potentially hazardous for ecosystem and human health [2,3]. Pymetrozine is a broad-spectrum insecticide, which has been used to control various insects by interfering with their nervous regulation of feeding behaviour [4,5,6]. At present, many countries and organizations have set their own maximum residue limit (MRL) of pymetrozine in various agricultural products. European Union (EU) and China Food and Drug Administration (CFDA) have established and revised MRL for pymetrozine in different products, which ranged 0.02–2.00 mg/kg [7,8]. These results suggest that the expected residual concentration of pymetrozine in agricultural and food samples is rather low, thus it is necessary to establish rapid, sensitive, and economical methods for determination of pymetrozine residues. Up to now, many methods have been published for detection of pymetrozine residues, including gas chromatography [4], gas chromatography-tandem mass spectrometry (GC-MS), high-performance liquid chromatography (HPLC) [9], liquid chromatography-tandem mass spectrometry (LC-MS/MS) [10,11], colorimetry [12], electrochemical method [13] and differential pulse polarography [14]. Although these methods exhibit good sensitivity, nevertheless, there are some drawbacks in their applications, such as laborious, complex procedures, and dependent on expensive equipment. To develop more simple, rapid, and low-cost methods for detection of pymetrozine residues is of great significance to food safety and human health.

Surface-enhanced Raman spectroscopy (SERS) is a vibrational spectroscopic technique, which combines the advantages of nanotechnology and Raman spectroscopy. Due to the advantages of high sensitivity, great selectivity, and low detection limit, SERS has been utilized for detection of chemical contaminants (such as pesticides, chemical and food additives) [15,16,17,18,19,20], mycotoxins [21], pathogens [22] in various products. In recent years, with the rapid development of nanomaterial technology, SERS was widely used for pesticide residue analysis. For instance, Jiao et al. used SERS method to quantify deltamethrin residue levels in wheat [23]. Hassan et al. synthesized flower-like silver nanoparticles (AgNPs) as SERS platform to detect methomyl, acetamiprid, and 2,4-D residue in green tea [24]. Likewise, Zhu et al. developed a micro-bowl arrays-based SERS sensor to detect thiram and methyl parathion residue on Chinese cabbage [25]. Meanwhile, Chen et al. developed the AgNPs based SERS sensor to measure phosmet in Oolong tea [26]. Moreover, Wang et al. [27] used the SERS-based method for trace detection of nitenpyram. Although there has been report on the application of SERS method for detection of pymetrozine, these studies only achieved the detection of pymetrozine in standard solution, and did not evaluate the feasibility for detection of pymetrozine in authentic food samples [28]. In general, the detection performances of SERS method primarily depend on the substrate used, and different substrates show variable detection effects. AgNPs are an easy to prepare and commonly used substrate due to their high surface plasmon resonances [29]. One of the main factors restricting the rapid development of SERS technology is the weak signal of the target analyte due to the weak affinity of metal nanomaterials to target molecules. However, when the surface of nanomaterials was functionalized by some special chemicals, the adsorption of the target analytes on nanoparticles could be improved, thus generating strong SERS signals [21,30,31,32].

In this paper, a SERS method by using AgNPs as substrate for detection of pymetrozine in apple and cabbage samples was developed. To improve detectability of AgNPs for pymetrozine, a simple strategy in which AgNPs surface was functionalized by decanethiol, was applied to enhance the adsorption of AgNPs with pymetrozine, and the functionalized AgNPs were used as substrate for SERS detection. The accuracy of the SERS method was evaluated by the detection of pymetrozine in spiked apple and cabbage samples, and the feasibility of SERS method in real sample detection was evaluated by the determination of pymetrozine residues in authentic cabbage samples, which was validated by LC-MS/MS. To the best of our knowledge, this is the first report dealing with the detection of pymetrozine residues in food samples by SERS method.

## 2. Materials and Methods

### 2.1. Materials and Reagents

Silver nitrate (AgNO_3_, 99.9%), trisodium citrate (dihydrate, 98%), sodium nitrate (NaNO_3_), anhydrous magnesium sulfate (MgSO_4_), sodium chloride (NaCl), methanol, acetonitrile, acetic acid, octanethiol, decanethiol, and decanedithiol were purchased from Shanghai Aladdin Bio-Chem Technology Co. Ltd. (Shanghai, China). C_18_ SPE cartridge (3 mL, 60 mg) was obtained from Agela Technologies (Tianjin, China) and bamboo charcoal (BC) was purchased from Shanghai Hinuo Charcoal Industry Co., Ltd. (Shanghai, China). Standard of pymetrozine (98.5% purity) was obtained from Dr Ehrenstorfer GmbH (Augsburg, Germany).

### 2.2. Fabrication of AgNPs and Functionalization with Different Chemicals

AgNPs were fabricated based on our previous study with some modifications [21]. In brief, 36.0 mg of AgNO_3_ was dissolved in 200.0 mL of Milli-Q water in a 500-mL clean flask, and was stirred (900× *g*) and heated (120 °C) to boiling with a heating magnetic stirrer (IKA RCT basic, IKA Inc., Breisgau, Germany). The solution was continuously boiled and stirred, and then 4.0 mL of trisodium citrate solution (1%, *m*/*v*) was rapidly added to the boiling solution. The mixed solution was kept stirring and boiling for 1 h and then cooled down to room temperature (25 °C). Finally, the AgNPs were prepared.

For functionalization of AgNPs, a constant volume of 100.0 μL different chemicals (octanethiol, decanethiol and decanedithiol) with a series of concentrations (1.0 × 10^−3^–1.0 × 10^−12^ mol/L) were added in 2.0 mL of AgNPs colloid and continuously shaken at 500× *g* for 25 min using a Multi-Tube Vortexer (MTV-100, Hangzhou Allsheng Instruments Co., Ltd., Hangzhou, China).

### 2.3. Characterization of Non-Modified and Modified AgNPs

UV-vis spectra of AgNPs and decanethiol modified AgNPs (M-AgNPs) in the range of 300–700 nm were recorded by an UV-vis spectrophotometer (TU-1901, Beijing Purkinje General Instrument Co., Ltd., Beijing, China), which were employed to characterize their optical properties. Transmission electron microscopy (TEM) images of AgNPs and M-AgNPs were obtained by a transmission electron microscope (JEM-1400 Plus, JEOL Ltd., Tokyo, Japan) with an acceleration voltage of 120 kV, which were used to characterize the morphological characteristics of the materials.

### 2.4. Preparation of Standard Solutions

Pymetrozine standard stock solution was prepared at a concentration of 1000 mg/L in methanol and stored at −18 °C in dark, and standard working solutions with various concentrations of 100.0, 50.0, 10.0, 5.0, 1.0, 0.5, 0.1, 0.05, 0.01, 0.005, and 0.001 mg/L were obtained by diluting the stock solution with methanol. The methanol was used as a blank control.

### 2.5. Sample Preparation

Apple and cabbage were purchased from a local supermarket in Guiyang, which were selected as representative food samples to confirm the feasibility of SERS method for detecting pymetrozine in food samples. All of these samples were tested to confirm the absence of pymetrozine by LC-MS/MS method [10,11]. The spiked apple and cabbage samples with adding levels of 0.2, 2.0, and 10.0 mg/kg were prepared by adding the pymetrozine working solution (the concentrations were 1.0, 10.0, and 100.0 mg/L, respectively) to these two homogenized samples. Five replicates were performed for each level, and the tests were repeated in two different days for an inter-day experiment. The extraction and cleanup procedures of the spiked samples were adapted the following steps.

Extraction of the spiked apple and cabbage samples was performed following a modified QuEChERS sample preparation method. Typically, the apple and cabbage samples were first homogenized adequately using a high-speed homogenizer, then 7.5 g of the homogenized samples were weighed into a 50-mL centrifuge tube, and 20.0 mL of acetonitrile (which contains 1% acetic acid) was also added to the tube, and then the samples in the tube were mixed. Subsequently, 3.0 g of NaCl and 3.0 g of MgSO_4_ were added to the tube, and the mixture was vortex-mixed again for 5 min, then was extracted by ultrasonic for 20 min, followed by centrifuging for 5 min at 6000× *g*, then 10.0 mL supernatant was taken into a 50-mL heart-shaped flask. The extraction was repeated twice, and the two extracts were mixed together. The extracts in the flask were evaporated at 40 °C under constant temperature. The evaporated residues were dissolved in 10.0 mL of methanol and transferred into a 15-mL centrifuge tube containing 500.0 mg of MgSO_4_ and 30.0 mg of BC sorbent. After mixing for 1 min, the solution was centrifuged for 5 min at 8000× *g*, and the supernatant was further purified by C_18_ SPE cartridge. The C_18_ SPE cartridge was first equilibrated with 5.0 mL of methanol and 5.0 mL of water, respectively. Then the supernatant was injected into the cartridge. After that the cartridge was washed with 8.0 mL of methanol/water (1:9, *v*/*v*). Finally, pymetrozine in the cartridge was eluted with 5.0 mL of methanol. Blank matrix solutions were prepared after the eluent was filtrated through a 0.22 μm filter. Matrix standard solutions with final concentration levels of 0.05, 0.1, 0.5, 1.0, and 5.0 mg/L were prepared by adding appropriate volumes of the standard working solutions to the blank matrix solutions.

### 2.6. SERS Measurement and Data Analysis

Prior to Raman measurement, a portion of 200.0 μL M-AgNPs was mixed with 200.0 μL of pymetrozine solution in a 1.5 mL centrifuge tube, and the mixed solution was stirred for 8 s, then 40.0 μL of NaNO_3_ solution (1 mol/L) was added to the tube and mixed for 8 s to facilitate AgNPs aggregation. The mixed solution was analyzed after these preparations. For SERS detection, the above solution was first sucked into a capillary with an inner diameter of 1 mm and then analyzed with a laser confocal microscopic Raman system (LabRAM HR, Horiba France SAS, Villeneuve, France) equipped with a high stable confocal microscope (BX41, Olympus Co., Center Valley, PA, USA), a 633 laser radiation, a grating of 600 grooves/mm, and a cooled CCD with 1024 × 256 pixels’ sensor. All Raman spectra were collected in the range of 300–2000 cm^−1^, and the acquisition time was 30 s with 2 accumulations. Each sample were repeated three times, and the mean value was used for analysis.

The Raman spectra were acquired and analyzed using a LabSpec 6 spectroscopy software suite (Horiba France SAS, Villeneuve, France). Two spectral preprocessing methods including denoising and baseline correction were performed to improve the signal to noise ratio and to minimize the interference of fluorescence.

### 2.7. The Correlation of SERS with LC-MS/MS

In order to verify the reliability of the proposed SERS method for determination of pymetrozine in real food samples, ten cabbage samples containing incurred residues were collected from a local farm in Guigyang in two different sampling sites (six samples in site one and four samples in the next one) were simultaneously analyzed by SERS and LC-MS/MS, and the correlation between the results of the two methods was evaluated. The pretreatments of the real samples were the same as the spiked samples described above. The analysis procedures of the spiked and real samples were the same as that of the standard solution, except that the 200.0 μL of eluate was used instead of 200.0 μL of pymetrozine standard solution.

LC-MS/MS analysis was performed on an AB Sciex 4000 QTrap LC-MS/MS system (Framingham, USA) equipped with an electrospray ionization source and was separated with XDB-C_18_ (4.6 × 150 mm id, 5 μm; Agilent Technologies, Santa Clara, CA, USA). The column temperature maintained at 40 °C. The mobile phase consisted of methanol and water (*v*/*v* = 7:3), and the analysis time was 6.0 min. The injection volume was 2 μL and the flow rate was 0.5 mL/min. Under these conditions, the retention time was 3.6 min.

Multiple reaction monitoring (MRM) with two mass conversions was performed to detect samples by triple quadrupole MS/MS with positive electrospray ionization mode. The optimization parameters were as follows: mass to charge ratio, 218.1 *m*/*z*, quantifier ion, 105.0 *m*/*z*, qualifier ion, 78.0 *m*/*z*, curtain gas, 25 psi, ion source gas 1, 50 psi and gas 2, 60 psi.

## 3. Results and Discussion

### 3.1. Characterization of AgNPs and M-AgNPs

**Figure 1:**
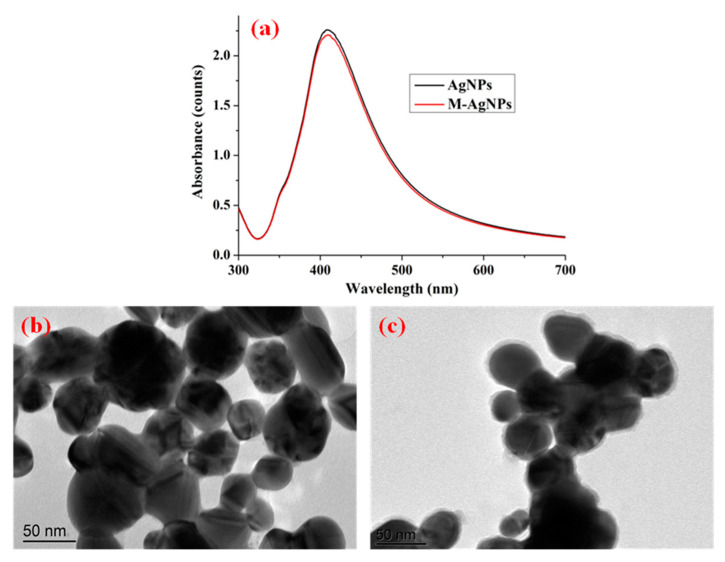
UV-vis spectra of AgNPs and M-AgNPs (**a**), TEM images of (**b**) AgNPs and (**c**) M-AgNPs3.2. SERS activity of different modified substrates.

UV-vis spectra and TEM images of the substrates were employed to evaluate their optical properties and morphological characteristics, which were also used to illustrate the successful synthesis and functionalization of AgNPs. Figure 1a shows the UV-vis spectra of AgNPs and M-AgNPs. As shown in Figure 1a, a maximum-absorption peak was centred at 412 nm for AgNPs, which corresponded to our previous study [21]. The absorption value of M-AgNPs was slightly lower than that of AgNPs, one possible reason is that decanethiol was tightly adsorbed on nanostructured silver surface. The narrow and strong absorption peak indicated excellent monodispersity and plasmon properties. Figure 1b,c present TEM images of the surface morphology of AgNPs and M-AgNPs. These images indicated that AgNPs were approximately spherical with an average diameter of 45 nm, and M-AgNPs had a shadow with an average thickness of 5 nm due to the fact that the decanethiol was adsorbed on the surface of AgNPs.

It is well-known that the high performance active substrate is of vital importance in SERS detection, and the intensity of SERS signal was directly related to the active substrate. In order to improve the SERS activity of AgNPs to pymetrozine, three kinds of chemicals including octanethiol, decanethiol, and decanedithiol were first applied to modify AgNPs, and the functionalized AgNPs were selected as active substrates. Pymetrozine with the concentration of 100 mg/L in methanol was selected as probe molecule to evaluate the SERS activity of different active substrates.

**Figure 2:**
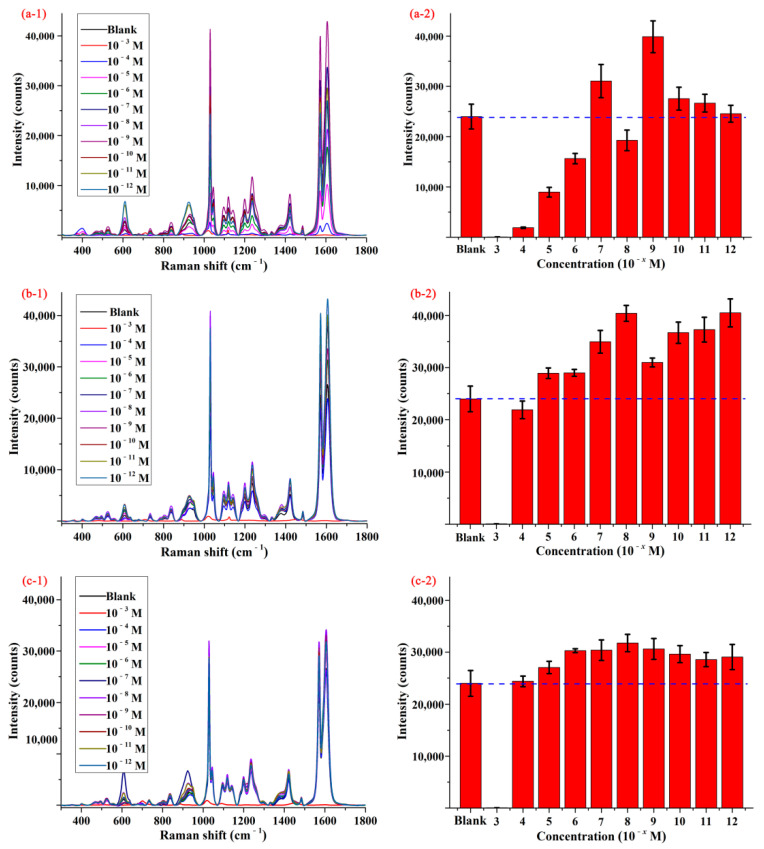
The average SERS spectra (**1**) and concentration-dependent intensity (**2**) of pymetrozine based on AgNPs modified by (**a**) octanethiol, (**b**) decanethiol, and (**c**) decanedithiol.

Figure 2(a-1–c-1) display the SERS spectra of pymetrozine obtained by using different substrates, and the spectrum of pymetrozine based on AgNPs was given as control. As shown in the figures, the characteristic peaks of pymetrozine were located at 610, 924, 1030, 1097, 1121, 1203, 1237, 1423, 1572, and 1606 cm^−1^, most of them (such as 1030, 1572, and 1606 cm^−1^) were consistent with previous study [28]. The intensities of the characteristic peak at 1572 cm^−1^ based on different substrates were given in Figure 2(a-2–c-2). As shown in these figures, compared with AgNPs, the functionalized AgNPs had a stronger enhancement effect on signal of pymetrozine, which means the sensitivity of AgNPs to pymetrozine can be improved by modifying its surface. The possible reason is that a layer of thiols was formed on the surface of AgNPs via the Ag-S bond between silver and thiol, and the chemical interaction between thiol and pymetrozine promoted the aggregation of pymetrozine on the surface of AgNPs, which can increase the adsorption capacity of the pymetrozine molecules on AgNPs [32,33,34]. An interesting phenomenon was that AgNPs functionalized by different chemicals with different concentrations presented a different enhancement effect on pymetrozine, and even some of them had negative impact on the SERS signal enhancement of pymetrozine. This was attributed to the following possible reasons: (i) the adsorption capacity of the thiol molecules is related to the length of carbon chain; and (ii) the high concentration of thiol may lead to aggregation of AgNPs. Comparing the results in Figure 2, AgNPs modified by decanethiol with the concentration of 1.0 × 10^−8^ mol/L were selected as active substrate for SERS detection.

### 3.2. SERS Activity of Pymetrozine on AgNPs and the M-AgNPs

**Figure 3:**
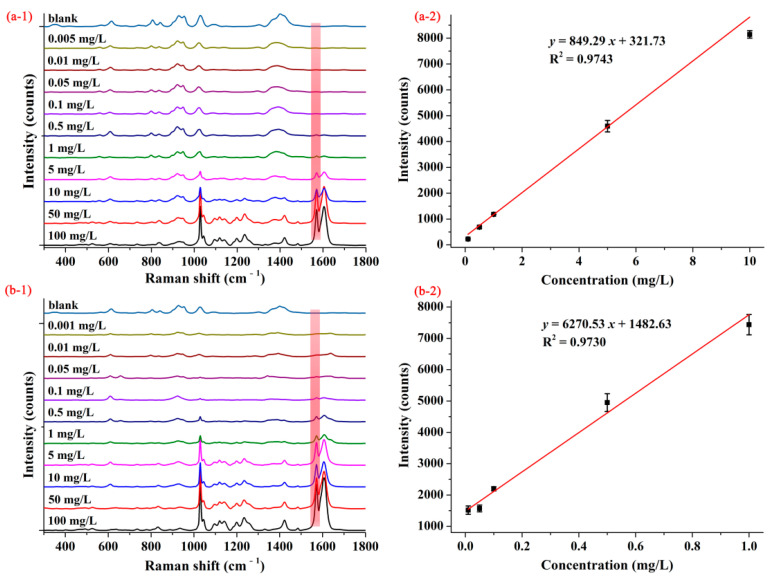
The average SERS spectra (**1**) and concentration-dependent intensity at 1572 cm^−1^ (**2**) of pymetrozine based on (**a**) AgNPs and (**b**) decanethiol M-AgNPs.

Figure 3(a-1,b-1) show the average SERS spectra of pymetrozine with concentrations from 0.001–100.00 mg/L based on AgNPs and M-AgNPs, respectively. As can be seen from Figure 3(a-1), when AgNPs were selected as SERS substrate, SERS intensity of pymetrozine at the characteristic peak decreased with the decrease of its concentration, if the concentration of pymetrozine went down to 0.1 mg/L, the SERS intensity of pymetrozine at the characteristic peak became very small, even no Raman peak can be observed. As shown in Figure 3(b-1), when M-AgNPs were used as the substrate, if the concentration dropped to 0.05 mg/L, the Raman characteristic peak was still observed. These results indicate that based on M-AgNPs substrate, SERS method can achieve higher detection sensitivity. The concentration-dependent SERS intensities for pymetrozine based on AgNPs and M-AgNPs are shown in Figure 3(a-2,b-2), respectively. As can be seen from these two figures, the SERS intensities had linear correlations with the concentrations of pymetrozine ranging from 0.10–10.00 mg/L and 0.01–1.00 mg/L, the linear relationships being *y* = 849.29 *x* + 321.73 with coefficient of determination (*R*^2^) of 0.9743 and *y* = 6270.53 *x* + 1482.63 with *R*^2^ of 0.9730, and the LOD was calculated as 0.05 and 0.01 mg/L, respectively. These results indicate that M-AgNPs showed a stronger ability to detect pymetrozine, thus it was selected as the SERS substrate in the subsequent studies.

### 3.3. Detection of Pymetrozine in Matrix Standard Solution and the Spiked Samples

**Figure 4:**
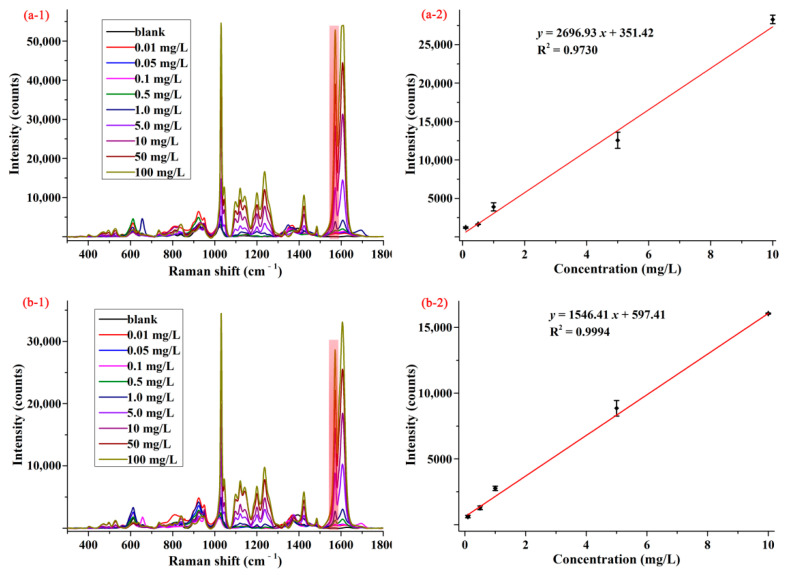
The average SERS spectra (**1**) and concentration-dependent intensity at 1572 cm^−1^ (**2**) of pymetrozine in (**a**) apple and (**b**) cabbage extracts.

Apple and cabbage samples were selected to assess the feasibility and practicability of SERS method for determination of pymetrozine in food products. In order to reduce the influence of sample matrixes, a modified QuEChERS sample preparation method was used to extract pymetrozine from apple and cabbage matrixes. The average SERS spectra of pymetrozine in apple and cabbage extracts are presented in Figure 4(a-1,b-1). As can be seen from these two figures, the characteristic peak (1572 cm^−1^) of pymetrozine in apple and cabbage extracts were the same as in methanol, and the intensity of pymetrozine was also positively associated with its concentration in the range of 0.01–100.00 mg/L. The concentration-dependent intensity at 1572 cm^−1^ for apple and cabbage extracts are shown in Figure 4(a-2,b-2). It was observed that the SERS intensity had linear correlation with the concentrations of pymetrozine in both apple extract and cabbage extract in the range of 0.1–10.0 mg/L, the linear relationship of which were *y* = 2696.93 *x* + 351.42 and *y* = 1546.51 *x* + 597.41, with the LOD value of 0.02 and 0.03 mg/L, the limit of quantification (LOQ) was 0.07 and 0.10 mg/L, respectively.

The spiked apple and cabbage samples were selected for the recovery tests, and the results of recovery tests are given in Table 1. As can be seen from Table 1, for apple, the recovery ranged from 70.40–100.35%, and all average recoveries of the three spiked levels were higher than 82.42%. The RSD of intra-day test was in the range of 7.17–12.18%, and the RSD of inter-day test ranged from 8.16–10.07%. Similar results were obtained in the recovery test of the spiked cabbage sample. These results indicated that the SERS method had a great potential for determination of pymetrozine in apple, cabbage, and other food samples.

In this study, the standard curve prepared by methanol could not be used for the recovery experiments of the spiked apple and cabbage samples. The main reason can be ascribed to the fact that the pymetrozine extracts from apple and cabbage samples usually contain various impurities, and the sample matrixes could affect the accuracy of SERS detection [21,35,36]. However, the matrix effect can be removed or reduced after a series of purification treatments, and matrix curves were used for apple and cabbage samples analysis.

### 3.4. The Validation of SERS with LC-MS/MS

**Figure 5:**
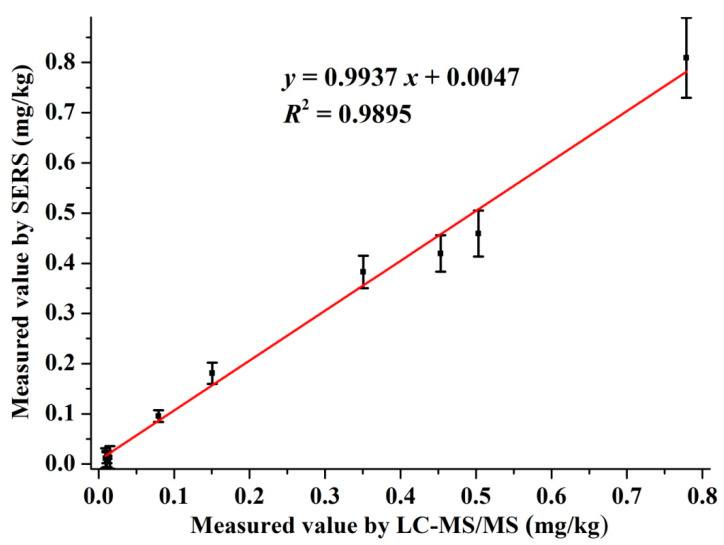
Correlation between SERS and LC-MS/MS for detection of pymetrozine in cabbage samples.

The residual amount of pymetrozine in the authentic cabbage samples were tested by SERS and LC-MS/MS simultaneously. The detection curve of pymetrozine by LC-MS/MS is given in Figure S1. The LOD and LOQ of the LC-MS/MS was 0.11 and 0.37 μg/L, respectively. The results and correlation analysis between the two methods are presented in Figure 5. As shown in Figure 5, the correlation equation of the two methods was *y* = 0.9937 *x* + 0.0047, and the *R*^2^ = 0.9895, which revealed the excellent correlation between SERS and LC-MS/MS. All these results indicated that the developed SERS method was suitable for monitoring pymetrozine residues in real cabbage samples, which has great potential for quantitative detection of pymetrozine in authentic food products.

## 4. Conclusions

In this study, a SERS method based on an easily prepared AgNPs substrate was first used for detection of pymetrozine in apple and cabbage samples. A simple method was proposed to improve the sensitivity of detection by modifying the AgNPs with decanethiol. The established method performed satisfactorily with the linear range of 0.01–1.00 mg/L and LOD of 0.01 mg/L in methanol. In addition, the method was successfully applied to detect pymetrozine in the spiked apple and cabbage samples, the LOD in apple and cabbage was 0.02 and 0.03 mg/L, and the recovery ranged 70.40–104.00%, with RSD below 12.18% and 10.33% for the intra-day and inter-day test, respectively. The results of SERS for the cabbage samples were in good agreement with those of LC-MS/MS, which confirmed the reliability of the proposed method for pymetrozine residue monitoring in real samples. The results indicated that the developed SERS method can be utilized to detect pymetrozine in apple and cabbage, which has great potential for determination of pymetrozine in other food samples.

## Figures and Tables

**Table 1:** The results of detecting pymetrozine in the spiked apple and cabbage samples.

Samples	Spiked Level (mg/kg)	Recovery (%)				RSD (%)		
		Day 1		Day 2		Intra-Day		Inter-Day
		Range	AVG	Range	AVG	Day 1	Day 2	D1 + D2
Apple	0.2	74.50–95.50	83.50	70.50–93.00	83.00	8.86	8.43	8.16
	2.0	72.45–100.35	87.15	70.40–96.90	83.61	10.90	10.07	10.07
	10	78.95–98.53	87.70	71.59–98.12	82.42	7.17	12.18	9.83
Cabbage	0.2	70.50–99.50	86.40	75.50–104.00	84.30	10.35	11.42	10.33
	2.0	73.40–99.25	83.34	75.20–98.50	86.84	9.66	9.55	9.24
	10	70.92–98.30	83.90	71.15–97.79	82.24	11.63	11.01	10.24

Note: D1 + D2 = Day 1 and Day 2, AVG = average.

## Data Availability

Data are contained within the article and supplementary material.

## Supplementary Materials

The following are available online at https://www.mdpi.com/article/10.3390/foods10081874/s1, Figure S1 Detection curve of pymetrozine by LC-MS/MS.
