# Supplementary figures and images for: A Sensitive SERS Method for Determination of Pymetrozine in Apple and Cabbage Based on an Easily Prepared Substrate
Source: Foods. 2021 Aug 13;10(8):1874. doi: 10.3390/foods10081874 (PMC8392414; doi:10.3390/foods10081874)

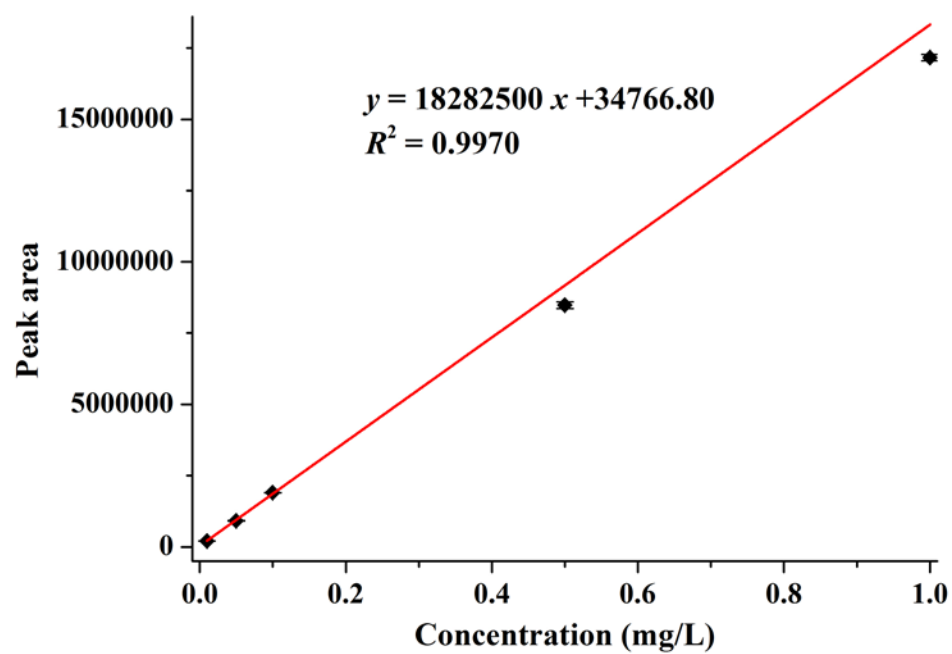

Fig. S1 Detection curve of pymetrozine by LC-MS/MS

Supplement: Supplementary file 1 [file foods-10-01874-s001.zip › foods-1309115-supplementary.pdf]
